# Assessment of semen quality of taxi drivers exposed to whole body vibration

**DOI:** 10.1186/s12995-022-00357-z

**Published:** 2022-08-03

**Authors:** Sirvan Zarei, Somayeh Farhang Dehghan, Mohammad Hossein Vaziri, Mohammad Ali Sadighi Gilani, Soheila Khodakarim Ardakani

**Affiliations:** 1 Workplace Health Promotion Research Center, School of Public Health and Safety, Shahid Beheshti University of Medical Sciences, Tehran, Iran; 2 Department of Urology, School of Medicine, Shariati Hospital, Tehran University of Medical Sciences, Tehran, Iran; 3 Department of Biostatistics, School of Medicine, Shiraz University of Medical Sciences, Shiraz, Iran

**Keywords:** Occupational exposure, Whole body vibration, Infertility, Taxi drivers, Semen quality

## Abstract

Whole body vibration (WBV) is a recognized occupational hazard for many workers such as drivers and operators of heavy and light machinery and exposure to it is accompanied by physical and mental repercussions. Only the limited studies have been done on the effects of vibration exposure on reproductive indices, however vibration exposure has been indicated as being a hazardous agents with the potential of being able to directly affect the reproductive system. Considering the importance of infertility, the present study was conducted with the aim of investigating semen quality among taxi drivers in Tehran and determine the effect of exposure to WBV on sperm parameters. The study population consisted of 70 taxi drivers and 70 office employee who attended infertility clinics for diagnostic purposes in the past year. The participants had to meet the entry criteria of the study while also being willing to recruit. Data was collected in the form of demographic questionnaires and general health questionnaires as well as sperm analysis conducted in an infertility clinic according to standard laboratory guidelines. The 8-hour time-weighted average (TWA) exposure to of WBV in automobile was measured as per ISO 2631-1. The TWA exposure to WBV among the taxi drivers and office employees were 0.697 ± 0.13 m/s^2^ and 0.068 ± 0.09 m/s^2^ respectively (*P* < 0.05). A statistically significant difference in total sperm count, progressive motility, non-progressive motility and total motility was observed between the taxi drivers and the office employees (*P* < 0.05). According to the univariate analysis of variance, exposure to WBV had negative effect on sperm concentration, progressive motility and normal morphology (*P* > 0.05); moreover exposure to WBV resulted in the larger effect size (B) on sperm parameters than the demographic variables (*P* > 0.05). Being taxi drivers increase the chance of the decreased semen quality (*P* > 0.05). It is difficult to draw definitive conclusions regarding the effects of WBV while intervening factors exist, such as psychological stressors, quality of sleep, background issues as well as environmental factors such as chemical pollutants (heavy metals) or ergonomic factors (body posture and working while sitting down).

## Introduction

Taxi drivers provide an essential service in the transportation of people in civil society, and maintaining the health and safety of drivers and passengers guarantee its efficient performance [1]. According to the statistics published by the municipal taxi department of the city of Tehran, over 100 thousand taxi drivers and around 80 thousand personal taxi service workers are currently occupied in the city [2]. Driving as an occupation has its own difficulties and may threaten the health of the drivers due to exposure to harmful occupational agents such as long term exposure to vibrations, lack of movement, being seated for an extended period of time, unsuitable body posture and frequent bending or turning movements which can all cause occupational disorders [3].

Whole body vibration (WBV) is one of the main pollutants caused by civil transportation systems and exposure to it can lead to serious harm in the long run. This exposure creates a complicated distribution of movements or vibrational forces inside the body and can harm health, reduce physical activity, cause discomfort and even lead to various disorders [4]. Barkhordari et al. measured WBV exerted on taxi drivers working in the city of Yazd, Iran. They found that the long working hours (5 to 12 hours a day) and lengthy traffic jams expose the drivers to considerable levels of WBV [5]. Humans are most sensitive to vibration in the 1 to 80 Hz range, but this is also dependent on velocity, wavelength and variance in acceleration [6]. Exposure of limbs or organs to vibrations at certain frequencies can be dangerous. The transfer of mechanical energy from the source of vibration to the body can induce musculoskeletal disorders, cause discomfort, reduce productivity and disrupt physiological and mental function in humans. WBV occurs when the individual in on a vibrating surface and the vibration is affecting areas in the body that are distant from the source [7]. This usually takes the form of mechanical vibrations from industrial machines or vehicles which are transferred to the entire body via the buttocks and legs [8]. Short term exposure to high intensity WBV may induce internal organ trauma while long term exposure to low intensity WBV may increase blood pressure and cause other stress related symptoms. Other better observed health effects of vibration include pain in the sciatic nerve, digestive disorders, issues involving the sex organs and hearing disorders [9].

The prevalence of infertility in Iran is 20.2%, which is estimated at 19.9% in urban areas and 22% in rural areas. This prevalence rate is almost high in the whole country and also, the statistics of the Iran Ministry of Health and Medical Education show that out of every five Iranian couples, there is one infertile couple in the country and about 80,000 infertile couples are added to the statistics every year [10]. Infertility is usually accompanied by numerous psychological and social problems. The financial, psychological, and physical challenges posed by assisted reproductive techniques are likely to have a greater impact on the infertile couple. The prevalence of infertility in the world, in addition to causing significant psychological problems for infertile couples, causes the economic burden to be healed and threatens the health and economy of the family. Infertility services are a part of medical services, a significant part of which is not covered by insurance, and incurs heavy costs for infertile individuals and couples [11, 12].

Not many studies have been conducted on the effects of direct exposure to vibrations on reproductive indices, yet vibration is reported to be an environmental stressor with high effect potential in regards to the reproductive system [4, 13, 14]. The prevalence among drivers occupied in industrial and agricultural jobs, of the inability to produce natural sperm compared to other occupations has been documented [15]. Similar reports have been made by Talamanca et.al regarding taxi drivers and those exposed to WBV [16]. A cohort study investigated the relationship between occupation, health, and semen quality among a total of 501 couples discontinuing contraception were followed for 1 year while trying to conceive. Their results showed that those with a history of exposure to WBV had altered sperm parameters, but vibration was not associated with semen quality [17]. Figà-Talamanca et al. assessed the association between the work exposures of professional drivers and their reproductive health, by studying a group of 201 taxi drivers in the city of Rome. The results showed that taxi drivers, compared to the controls, had a significantly lower prevalence of normal sperm forms (45.8% vs. 64.0%) [16].

The daily exposure of taxi drivers to these vibrations is inevitable, although improvements to suspension systems of automobiles have reduced vibrations to the lowest levels in recent years [18]; it is still a cause of environmental pollution. It is important to evaluate the exposure level and health side effects in regards to harmful environmental factors such as whole body vibration. Yet few studies have been done in this particular field and especially among taxi drivers. Knowledge of the effect of WVB on the quality of sperm is necessary for this particular occupation. Thus, the aim of the present study is to assess the quality of semen among taxi drivers working in the city of Tehran who referred to a fertility clinic during 2020–2021 and also it aimed to determine the effect of exposure to WBV on sperm parameters.

## Methods

The study population consisted of 70 taxi drivers and 70 office employee who referred to a fertility clinic during 2020–2021. The participants had to meet the entry criteria of the study while also being willing to recruit. The participants were required to be 20 to 50 years of age with at least 2 years of employment [19]. Those who had used steroids, prednisolone, testosterone (prior to clinic referral), anti-oxidants (such as selenium), vitamins B, E or C (prior to clinic referral) or bodybuilding supplements (prior to clinic referral) were excluded. Participants who had a familial history of infertility or organic disorders affecting reproductive performance such as diabetes, kidney disorders, angina pectoris, heart disorders, arterial blood pressure, disorders of the pituitary gland, CPOD, testicular infection, testicular inflammation, varicocele or a history of chemo or radio therapy [19] and also people who change the car during the past year and their car had the serious damage during the past year were deemed to meet exclusion criteria. The taxi driver drove at least 8 hours a day or two shifts of 4 hours and 4 days a week. In the case of administrative staff, the requirement to enter the study was to have at least 1 year of work experience with a maximum of 8 hours of daily work and at least 4 hours of daily sitting work. Participants who did not complete the questionnaires or were unwilling to continue at any stage in the study and excluded from the study. The written informed consent was obtain from all subjects before their participation. It has been approved by the ethics committee of Shahid Beheshti University of Medical Sciences (IR.SBMU.PHNS.REC.1398.164).

Semen samples were taken for sperm analysis (Spermogram) via masturbation into a sterile plastic container after 3 days of celibacy. The samples were collected at a fertility clinic in the city of Tehran in order to prevent the effects of heat, contamination by secretions of the female reproductive organ and faster testing. Sperm parameters such as concentration, pH, color, viscosity, motility, total sperm count, and morphology were measured as per the guidelines of the World Health Organization (WHO) [20] using computer-assisted sperm analysis (CASA). This involves the use of a phase contrast microscope (NikonTM Eclipse E-200, Japan) connected to a camera (BaslerTM, A312FC, Germany) aided by software (SCA Microptic S.L., Spain). According to the WHO in 2010, a normal semen analysis conforms to the following: the sample must contain a minimum volume of 2 ml, semen must have a pH higher or equal to 7.2, the sperm concentration (number of sperm per milliliter) must be equal or above 15 million/ml, total sperm count must be larger than 39 million per ejaculation, at least 40% of the sperm must have movement until 1 hour after ejaculation, a minimum of 32% of sperm must have rapid progressive movement and 4% must be have normal morphology as per Kruger’s criteria [20].

**Fig. 1:**
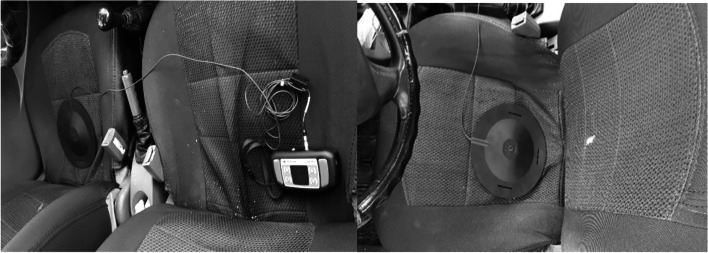
Measurement of whole body vibration among the study population

The next stage involved the measurement of vibration acceleration in three axes via a calibrated WBV meter (Bruel and Kjaer model 4447) which has a thin disk and a plastic cover and is placed on the buttocks area of the driver seat (Fig.1). Measurements were taken in m/s^2^ for 30 minutes and in all three axes (X, Y and Z) as per the ISO 2631 standard [21] when participants were driving certain ways around the infertility clinic. The overall vector sum and the acceleration level were calculated using the relevant equations. The root mean square (RMS) of acceleration for each of the axes was obtained using Eq. 1 (measurement time of 30 minutes) from the exposure group (cars of taxi drivers) and the control group (cars of office employees).

1 $${a}_{w=}{\left[\frac{\mathbf{1}}{\boldsymbol{T}}\int_{\mathbf{0}}^{\boldsymbol{T}}{{\boldsymbol{a}}_{\boldsymbol{w}}}^{\mathbf{2}}\left(\boldsymbol{t}\right)\boldsymbol{dt}\right]}^{\frac{\mathbf{1}}{\mathbf{2}}}$$

In the above equation, *a*_*w*_ is the root mean square (RMS) frequency-weighted acceleration in each axis, *a*_*w*_ *(t)* is the frequency-weighted acceleration at the moment of *(t)* and *T* is the measurement duration. The vibration acceleration vector sum was calculated using Eq. 2 for each of the three axes in m/s^2^.

2 $${a}_{v=}\sqrt{{\left(\mathbf{1}.\mathbf{4}{\boldsymbol{a}}_{\boldsymbol{wx}}\right)}^{\mathbf{2}}+{\left(\mathbf{1}.\mathbf{4}\ {\boldsymbol{a}}_{\boldsymbol{wz}}\right)}^{\mathbf{2}}+{\left(\mathbf{1}.{\mathbf{a}}_{\boldsymbol{wz}}\right)}^{\mathbf{2}}}$$

Here, *a*_*v*_ is the vector sum of the RMS frequency-weighted accelerations while *a*_*x*_, *a*_*y*_ and *a*_*z*_ represent the RMS frequency-weighted accelerations in the specific direction. The daily 8-hour frequency weighted exposure to vibration acceleration is determined using Eq. 3.

3 $$A(8)={\boldsymbol{a}}_{\boldsymbol{v}}\sqrt{\frac{\boldsymbol{T}}{{\boldsymbol{T}}_{\mathbf{0}}}}$$

In the above equation, *a*_*v*_ is the frequency-weighted acceleration during exposure, *T*_*0*_ is the average exposure duration (8 hours for taxi drivers and 2 hours for office employees) and *A(8)* is the 8-hour equivalent RMS frequency-weighted acceleration in m/s^2^ [22].

Participants also filled the demographic questionnaire (age, driving experience, body mass index, education, smoking habits, and physical exercise), the general health questionnaire.

The General Health Questionnaire (GHQ) was devised by Goldberg and Hillier in 1979 [23] with 28 items and four aspects of distress which include somatic symptoms, anxiety and insomnia, social dysfunction and severe depression. Taghavi et al. assessed the viability of the Farsi translation of the GHQ questionnaire and reports a reliability of 0.70 as per the Test-Retest method, 0.90 as per Cronbach’s alpha and 0.93 as per the Split-Half method [24]. A 4-point Likert scale is used for scoring (0, 1, 2 and 3) with a maximum obtainable score of 84. Scores between 0 and 22 are indicative of health (none or minimal distress) and scores above 22 are indicative of psychological distress and are further divided into 3 ranges namely weak, medium or extreme distress.

After data was collected in the form of questionnaires, vibration measurements and sperm analysis, data distribution normality was determined using the Shapiro-Wilk test. Various tests including t-test, ANOVA, crosstab, Mann-Whitney, Chi-square, Pearson and Spearman test were used to compare normal and non-normal quantitative and qualitative data in the control and the exposure groups. Descriptive statistics are presented in the form of mean, median, standard deviation and interquartile range. The relationship between exposure intensity and reproductive parameters was determined using partial correlation and univariate analysis of variance. Data analysis was performed using SPSS v26 (Chicago, Illinois, USA) with a *P*-value bellow 0.05 considered to be statistically significant.

## Results

The results of the Shapiro–Wilk test revealed a normal distribution of data for age, physical activity and general health score with the remaining data being non-normal in its distribution. Table 1 shows descriptive statistics for demographic variables among the taxi drivers and the office employees. Mean age of participants in the taxi drivers and office employees were 36.84 ± 5.24 years and 37.70 ± 5.20 years, respectively (*P* = 0.832). Amount of previous driving experience was 8.98 ± 4.01 years among the taxi drivers and 8.40 ± 2.40 years among the office employees (*P* = 0.754). Additionally, among the taxi drivers, 33 (47.1%) were non-smokers, 17 (34.3%) were frequent smokers and 20 (28.6%) were infrequent smokers. As for the office employees, 33 (47.2) were non-smokers, 16 (22.8) were frequent smokers and 21 (30.0) were infrequent smokers. Among the taxi drivers, 44 (62.9%) had no light physical exercise, 20 (28.6%) had medium physical exercise (75 to 150 minutes) and 8 (8.6%) had extreme physical exercise (75 to 150 minutes or more). Among the office employees, 47 (67.2) had no light physical exercise, 21 (30.0) had medium physical exercise and 2 (2.8) had extreme physical exercise. The average general health scores obtained by the taxi drivers and office workers were 69.58 ± 6.98 and 70.20 ± 6.85, respectively. Number of participants with a BMI in the range of 18.5–24.9 was 42 (60%) for both the taxi drivers and the office employees.

**Table 1:** Descriptive statistics of demographic characteristics among the participants

Group		Taxi drivers (*n* = 70)	Office employees (*n* = 70)	*P*-value
Variable				
Age (years) -Mean (SD)		36.84 (5.24)	36.6 (5.2)	0.832*
Driving experience (years) -Mean (SD)		8.98 (4.01)	8.40 (2.40)	0.754**
GHQ -Mean (SD)		69.58 (6.98)	70.20 (6.85)	0.686*
Education N(%)	Primary Education	16 (22.9)	12 (17.2)	0.948***
	High School Diploma	21 (30.0)	23 (32.8)	
	Associate Degree	5 (7.1)	7 (10)	
	Bachelors	25 (35.7)	26 (37.2)	
	Masters	3 (4.3)	2 (2.8)	
Smoking Habit N(%)	Yes, Routinely	17 (24.3)	16 (22.8)	0.988***
	No	33 (47.1)	33 (47.2)	
	Yes, Intermittently	20 (28.6)	21 (30.0)	
Physical Exercise (per week) N(%)	No Exercise/Light Activity	44 (63.9)	47 (67.2)	0.642***
	Medium/75 to 150 min	20 (28.6)	21 (30.0)	
	Intense/> 75 to 150 min	8 (8.6)	2 (2.8)	
BMI (Kg/m^2^) N(%)	18.24–5.9 (Normal)	42 (60)	42 (60)	1.000***
	29–25.9 (Overweight)	28 (40)	28 (40)	

*GHQ* General Health Questionnaire, *BMI* Body mass index

* T-test, **Mann-Whitney, ***Chi-Square Pearson

Table 2 shows the WBV measurements among taxi drivers and office employees. The time-weighted average exposure to whole body vibration (m/s^2^) obtained for the taxi drivers and the office employees were 0.69 ± 0.13 m/s^2^ and 0.068 ± 0.09 m/s^2^, respectively. This places the taxi drivers and office employees in the medium and low health risk range, respectively [21].

**Table 2:** Time-weighted average exposure to whole body vibration (m/s^2^) among the studied groups

Group	Mean (SD)	Max	Min	Median (IQR)	*P*-value*
Taxi drivers	0.69 (0.13)	1.06	0.47	0.66 (0.18)	0.001
Office employees	0.06 (0.09)	0.09	0.05	0.70 (0.01)	

*Mann-Whitney

The results of the semen quality analysis are presented in Table 3 for both the groups. The statistical analysis of the semen parameters reveals that a statistically significant difference was observed between the two groups regarding total sperm count, progressive motility, non-progressive motility and total motility (*P* < 0.05). However, this was not the case for other parameters namely volume, pH, concentration, immotile sperm, regular morphology, irregular morphology, color and viscosity (*P* > 0.05).

**Table 3:** Results of the semen analysis on the studied groups

Quantitative Variable			Mean	Median	SD	IQR	*P*-value
Volume (ml)		Taxi drivers	3.68	3.25	1.98	2.88	0.142**
		Office employees	3.97	3.85	1.43	2.47	
pH		Taxi drivers	7.74	7.80	0.166	0.10	0.329**
		Office employees	7.56	7.80	0.711	0.10	
Concentration (number of sperm per milliliter) (10^6^ml per ml)		Taxi drivers	52.74	50.00	46.02	59.25	0.103**
		Office employees	66.62	65.00	48.53	64.25	
Total number of sperm in the entire ejaculate (10^6^ ml)		Taxi drivers	154.60	109.15	151.60	160.5	0.006**
		Office employees	321.65	209.00	433.84	239.4	
Total Motility (PR + NPR) (%)		Taxi drivers	40.28	42.05	22.61	35.1	0.002*
		Office employees	55.77	58.10	22.85	30.8	
Progressive Motility (%)		Taxi drivers	19.32	15.00	14.25	22.1	0.010**
		Office employees	27.31	27.00	14.25	18.5	
Non-Progressive Motility (%)		Taxi drivers	22.55	20.00	14.54	18.75	0.037**
		Office employees	28.45	26.00	13.83	13.85	
Normal Morphology (%)		Taxi drivers	2.76	3.00	1.77	3.00	0.766**
		Office employees	2.86	3.00	1.63	2.00	
Abnormal Morphology (%)		Taxi drivers	96.29	97.00	1.62	2.00	0.869**
		Office employees	97.13	97.00	1.63	2.00	
Qualitative Variable		Number (%)				0.304***	
		Taxi drivers		Office employees			
Color	Light Grey	34 (48.6)		19 (27.1)			
	Dark Grey	28 (40.0)		40 (57.1)			
	Yellow Grey	5 (7.2)		5 (7.2)			
	Bright Yellow	2 (2.8)		5 (7.2)			
	Milky	1 (1.4)		1 (1.4)			
Viscosity	Normal	66 (94.3)		58 (82.8)		0.089***	
	Abnormal	4 (5.7)		12 (17.2)			

*T-test, **Mann-Whitney, ***Chi-Square

In order to model and analyze the relationships between the variables, univariate analysis of variance was done. The effect of occupation and demographic variables on some semen parameters including concentration, progressive motility, immotile sperm and normal morphology have been presented in Table 4. In order to see the effect of each variable and its role in the regression model, one must refer to the standardized coefficient column (Beta/B). Those variables which have a higher standard coefficient have a more prominent role in predicting the dependent variable. As per Table 4, group type (Taxi drivers) had the negative effect (B = -47.141) on sperm concentration (*P* = 0.102), with the taxi drivers being 47.14-fold more likely to have lower sperm concentration compared to the office employees. Similar results (B = -15.846) were obtained regarding the effect of the group type on progressive motility (*P* = 0.071). As for immotile sperm, the likelihood of this variable being higher in the taxi drivers compared to the office employees was 5.26-fold greater (B = 5.261, *P* = 0.724). The group type had the decremental effect (B = -1.559) on sperm normal morphology with the probability of the taxi drivers having a higher rate of normal morphology sperm being 1.55-fold lower than office employees (*P* = 0.442). Exposure to WBV had negative effect on sperm concentration (B = -49.376; *P* = 0.249) progressive motility (B = -13.117; *P* = 0.314) and normal morphology (B = -2.631; *P* = 0.121); moreover exposure to WBV resulted in the larger effect size (B) on sperm parameters than the demographic variables (*P* > 0.05).

**Table 4:** Results of the univariate analysis of variance of some sperm parameters for all participants

Variable		Sperm Concentration			
		*P*-value	95% Confidence Interval		Exp (B)
			Lower Bound	Upper Bound	
Age		0.162	− 0.552	3.244	−1.346
BMI		0.758	−5.862	4.285	− 0.789
Whole Body Vibration		0.249	−56.597	32.214	−49.376
Smoking Habit	Yes, Routinely	0.290	−16.597	41.652	−14.528
	No	0.835	−20.146	24.873	2.363
Physical Exercise	No Exercise/Light Activity	0.551	−50.402	27.075	−11.664
	Medium	0.721	−47.847	33.244	−7.302
Group	Taxi drivers	0.102	−103.783	9.501	−47.141
Variable		Progressive Motility			
		*P*-value	95% Confidence Interval		Exp (B)
			Lower Bound	Upper Bound	
Age		0.399	−0.483	0.981	−0.399
BMI		0.914	−1.456	1.624	−0.084
Whole Body Vibration		0.314	−14.620	38.853	−13.117
Smoking Habit	Yes, Routinely	0.581	−10.581	5.964	−2.308
	No	0.285	−3.185	10.688	3.751
Physical Exercise	No Exercise/Light Activity	0.197	−8.087	19.514	−7.713
	Medium	0.186	−8.047	20.585	−7.302
Group	Taxi drivers	0.071	−33.104	1.441	−15.846
Variable		Immotile Sperm			
		*P*-value	95% Confidence Interval		Exp (B)
			Lower Bound	Upper Bound	
Age		0.745	−0.831	1.157	0.163
BMI		0.745	−4.197	1.064	1.567
Whole Body Vibration		0.894	−46.908	40.984	2.962
Smoking Habit	Yes, Routinely	0.894	−15.191	13.060	1.066
	No	0.333	−17.650	6.039	−5.805
Physical Exercise	No Exercise/Light Activity	0.620	−25.202	15.098	5.052
	Medium	0.973	−24.388	20.673	7.302
Group	Taxi drivers	0.724	−24.207	34.728	5.261
Variable		Normal Morphology			
		*P*-value	95% Confidence Interval		Exp (B)
			Lower Bound	Upper Bound	
Age		0.006	0.028	0.164	−0.096
BMI		0.135	−0.316	0.043	−0.136
Whole Body Vibration		0.121	−3.638	5.631	−2.631
Smoking Habit	Yes, Routinely	0.237	−1.542	0.387	−0.577
	No	0.638	−0.616	1.000	0.192
Physical Exercise	No Exercise/Light Activity	0.441	−0.839	1.911	−0.536
	Medium	0.495	−0.940	1.931	0.495
Group	Taxi drivers	0.442	−3.570	0.452	−1.559

## Discussion

Based on the findings of the present study, taxi drivers spend 4 to 14 hours a day driving, while this is only 2 to 4 hours a day for office employees. This is compounded by the low quality and old age of vehicles used as taxis in the country of Iran and specifically the city of Tehran. The drivers of these vehicles are averagely exposed to whole body vibration for 8 hours each day. This may lead to serious health issues and places this particular job in the list of high-risk occupations which involves multiple harmful physical agents. The results of the Mann-Whitney statistical test show a statistically significant difference in the WBV exposure between the taxi drivers and the office employees (*P* < 0.05). This issue can be justified by the vehicles suspension system, type of tire, quality of roads, increased performance and age of the taxi vehicle as well as longer periods in traffic, type of car and even driving habits [25]. The WBV acceleration of the taxis measured in the present study (0.69 m/s^2^) were similar to those measured by Soleimanian et al. in their study on inner-city taxi drivers (0.60 m/s^2^) [26]. The effect of WVB on the organ being vibrated is not destructive in the short run and only causes reduced performance in the individual. People who are exposed to high amplitude vibrations for extended periods of time each day will suffer adverse effects in the long run. The effect of low amplitude vibration on the human body is not well understood. The threshold of adverse effects due to vibration also varies among different individuals which makes it impossible to define threshold limits and sensitivity to vibration [27].

According our finding, a statistically significant difference in total sperm count, progressive motility, non-progressive motility and total motility was observed between the taxi drivers and the office employees (*P* < 0.05). Statistical analysis and modeling of the data presented in Table 4 shows being taxi drivers had the negative effect on sperm concentration, progressive motility and normal morphology(*P* > 0.05). As for immotile sperm, the likelihood of this variable being higher in the taxi drivers compared to the office employees was 5.26-fold greater (*P* = 0.724). Exposure to WBV had negative effect on sperm concentration progressive motility and normal morphology (*P* > 0.05) and non-significant positive on immotile sperm percentage. Vaziri et al. (2011), also claim a relationship between type of occupation and quality of sperm and state that the lowest mean sperm motility they observed was among those working in the transportation industry [28]. A future cohort study by Eisenberg et al. (2015) found that 23% of participants had exposure to WBV vibrations while 27% had exposure to noise in their occupational environment. Their regression analysis revealed that mean ejaculate concentration, total sperm count and DNA fragmentation index was lower in the control group although similarly to the present study, this was not statistically significant [17]. Al-Azzawi et al. (2018) investigated the effect of mechanical vibration on sperm activity in humans in laboratory conditions. They found that vibration had caused a significant increase in the rate of fast progressive motility (grade A), an insignificant increase in slow progressive motility and an insignificant reduction in the number of immotile sperm. They found no significant change in sperm morphology and total sperm count. They concluded that simple vibration of the semen sample for 20 minutes increases overall sperm activity with a considerable increase in the percentage of highly active fast progressive sperm [29]. In another study, Jurewicz et al. (2014) looked at the relationship between exposure to occupational factors and semen quality parameters and found that occupational factors may affect the quality of semen. Exposure to environmental or occupational pollutants such as noise, low physical activity, sitting for long periods of time, poly vinyl chloride and exposure to sound and noise during work were accompanied by reduction in sperm parameter indices in humans. They found a significant inverse relationship between occupational exposure to vibration and reduced sperm motility and increased DNA fragmentation [30].

The findings of the present study showed the negative effect of age, BMI, smoking and no physical activity on the studied sperm parameters like sperm concentration, progressive motility and normal morphology (Table 4); however these effect were statistically non-significant. The observation that with older age most sperm parameters are reduced and thus conception is unsuccessful due to damaged sperm is somewhat congruent with the results of Babakhah et al. (2017) and Gao (2006) [31, 32]. Cohen-Bacrie et al. reports that higher BMI was accompanied by reduced semen quality, ejaculate volume, sperm concentration and motility as well as increased abnormal morphology [33]. They stated that obesity may have led to reduced supply of sperm and faster transfer in the epididymis [33]. Mínguez-Alarcon et al. are in agreement with the present study and report no significant relationship between the range of physical exercise (less than 5 hours and between 0 to 40 hours a week) and sperm parameters among young and healthy Spanish men [34]. Ghahremanei et al. report that smoking one more cigarette per day reduces sperm count by 800 thousand and reduces motility by 1%. They also report lower ejaculate volume, sperm count and sperm motility among smokers compared to non-smokers [35]. Asare-Anane et al. has also stated that smoking can reduce ejaculate volume, sperm viability, motility, morphology and concentration [36]. No statistically significant difference in GHQ scores were observed between the taxi drivers and office employees (*P* > 0.05). Since the GHQ scores of both groups were higher than 24, it can be concluded that participants in both groups were in bad health status and thus immediate remedial measures must be taken. This is not surprising considering the long working hours, constant exposure to mental tension due to interactions with customers, bad posture during work, difficult economic conditions and mental stress experienced by them [37].

Considering the small number of studies, especially those with a focus on the effects of occupational exposure to vibration on reproductive indices, it is difficult to arrive at conclusions especially considering the contradictory reports and findings in the literature. Research on the potential risk of mechanical vibrations on the reproductive system is mostly limited to experimental, clinical and epidemiological studies involving lab animals and men occupied in industry and transportation, while also looking at the effect on libido [4, 38]. None the less, the negative effects of chronic exposure to WBV on the reproductive system in men especially with regards to sitting occupations are clear [30]. Despite its limitations, the present study has shown how exposure to WBV can affect semen parameters. Vibration has been cited as being an environmental stressor with the potential of affecting the reproductive system in men [13]. This is because of the high prevalence of reported cases of sperm disorders among drivers occupied in industrial and agricultural jobs, especially taxi drivers [16]. Despite the limited availability of credible sources, it seems that the potential mechanism of effect involved in the impact of vibration on the reproductive system is via hormone and enzyme levels, disruption in blood circulation in the testicular tissue, atrophy and changes in temperature [29]. In vitro studies have noted increased mobility of sperm in response to short term exposure to vibration without changes to morphology or sperm count [39].

One of the limitations of the present study is that other environmental factors such as air or noise pollution and work-related stressors have not been accounted for, as these can also influence results. Additionally, the samples were taken from inner city drivers and employees working in the city and so the results should not be extended to include other statistical populations (suburban drivers). Studies with larger sample sizes must be carried out at the national level in order to extend these conclusions to others. Moreover, it has been suggested that for future study, those undergoing fertility treatment may be more interested to be enrolled in the study and this may impact the generalizability of the findings.

## Conclusion

The present study aimed to assess semen quality among taxi drivers in Tehran and determine the effect of exposure to WBV on sperm parameters. A statistically significant difference in total sperm count, progressive motility, non-progressive motility and total motility was observed between the taxi drivers and the office employees. According to the univariate analysis of variance, exposure to WBV had a decremental effect on the most of sperm parameters, but these effects were not statistically significant. It is difficult to draw definitive conclusions regarding the effects of WBV while intervening factors exist, such as psychological stressors, quality of sleep, background issues as well as environmental factors such as chemical pollutants (heavy metals) or ergonomic factors (body posture and working while sitting down). A follow-up study is highly suggested considering the limitation of the presents study noted above.

## Data Availability

Not available.
